# Immunosuppressive Amino-Acid Catabolizing Enzymes in Multiple Sclerosis

**DOI:** 10.3389/fimmu.2020.600428

**Published:** 2021-01-20

**Authors:** Jorge Correale

**Affiliations:** Department of Neurology, Fleni, Buenos Aires, Argentina

**Keywords:** multiple sclerosis, amino acids, tryptophan, arginine, mammalian target of rapamycin, general control nonderepressible 2 kinase, kynurenine

## Abstract

Multiple sclerosis (MS) is a chronic inflammatory demyelinating disease that affects the central nervous system. Although the pathogenesis of MS is not yet fully elucidated, several evidences suggest that autoimmune processes mediated by Th1, Th17, and B cells play an important role in the development of the disease. Similar to other cells, immune cells need continuous access to amino acids (AA) in order to maintain basal metabolism and maintain vitality. When immune cells are activated by inflammation or antigenic signals, their demand for AA increases rapidly. Although AA deprivation itself may weaken the immune response under certain conditions, cells also have AA sensitive pathways that can activate intense alterations in cell metabolism based on changes in AA levels. Several data indicate that cells expressing enzymes that can degrade AA can regulate the functions of antigen-presenting cells and lymphocytes, revealing that the AA pathways are essential for controlling the function, and survival of immune cells, as well as immune cell gene expression. Basal AA catabolism may contribute to immune homeostasis and prevent autoimmunity, while increased AA catalytic activity may enhance immune suppression. In addition, there is increasing evidence that some downstream AA metabolites are important biological mediators of autoimmune response regulation. Two of the most important AA that modulate the immune response are L-Tryptophan (Trp) and L-Arginine (Arg). Tryptophan is catabolized through 2,3-dioxygenase (TDO) and indoleamine 2,3-dioxygenase (IDO) 1 and IDO2 enzymes, while three other enzymes catabolize Arg: inducible nitric oxide synthetase (iNOS), and two arginase isoforms (ARG1, ARG2). Genes encoding IDO, iNOS and ARG are induced by inflammatory cues such as cytokines, a key feature that distinguishes them from enzymes that catabolize other AA. Evidence suggests that AA catabolism is decreased in MS patients and that this decrease has functional consequences, increasing pro-inflammatory cytokines and decreasing Treg cell numbers. These effects are mediated by at least two distinct pathways involving serine/threonine kinases: the general control nonderepressible 2 kinase (GCN2K) pathway; and the mammalian target of rapamycin (mTOR) pathway. Similarly, IDO1-deficient mice showed exacerbation of experimental autoimmune encephalomyelitis (EAE), increased Th1 and Th17 cells, and decreased Treg cells. On the contrary, the administration of downstream Trp metabolite 3-HAA, inhibits Th1/Th17 effector cells and promotes Treg response by up-regulating TGF-β production by dendritic cells, thereby improving EAE. Collectively, these observations stand out the significance of AA catabolism in the regulation of the immune responses in MS patients. The molecules related to these pathways deserve further exploration as potential new therapeutic targets in MS

## Introduction

Multiple Sclerosis (MS) is a chronic inflammatory disease of the central nervous system (CNS) leading to demyelination and neurodegeneration. Although its pathogenesis is not yet fully understood, there is considerable evidence to suggest that MS is an autoimmune disease mediated by auto-reactive T and B cells in early stages, while innate immunity (monocytes and microglia cells) contribute to further axonal degeneration, neuronal loss and progression of the disease (1). On the other hand, FOXP3^+^ regulatory T cells (Treg cells) and IL-10 secreting type 1 regulatory T cells (Tr1), regulate the activity of effector T cells, accordingly, deficits in Treg cells and Tr1 cells have been described in MS (2–4). Thus, the balance between effector and regulatory cells controls MS disease activity (5–7).

Similar to other cells, immune cells need continuous access to amino acids (AA) in order to maintain basal metabolism and remain viable. When immune cells are activated by inflammation or antigenic signals, their demand for AA increases rapidly, thereby supporting higher energy and anabolic demands (8). Notably, these metabolic changes that arise are intricately linked to their differentiation status. Limited access to AA during immune cell activation may compromise immune responses by inhibiting immune cell division, differentiation, maturation, migration, and acquisition of new effector functions (9). An increasing body of data shows that cells express enzymes that degrade AA and modulate antigen presenting cells (APC) and lymphocyte function. Basal AA catabolism may contribute to immune homeostasis, preventing autoimmunity, whereas elevated AA catalytic activity may reinforce immune suppression, promoting pathogen persistence and chronic infections. Furthermore, accumulating evidence shows that several downstream AA metabolites are important biological mediators of autoimmune response regulation (10, 11). Although AA deprivation per se may attenuate immune response under certain conditions, cells also possess AA-sensing pathways that trigger changes in cell metabolism in response to depletion of specific AA in the microenvironment (12). Indeed, some AA catabolic pathways have become critical checkpoints of immunity (13). To date, the most studied are the pathways regulating the L-tryptophan (Trp) and L-arginine (Arg) catabolism.

L- Tryptophan is catabolized by Trp 2,3-dioxygenase (TDO) and two isoenzymes of indoleamine 2,3-dioxygenase (IDO1, and IDO2). Immune regulation by IDO1 is thought to be mediated by Trp deprivation, which directly impacts immune cell survival and function, or by downstream immunosuppressive Trp metabolites (11, 14, 15). L-Arginine is catabolized by two arginase isoforms (ARG1, ARG2) and by inducible nitric oxide synthetase (iNOS). Genes encoding IDO, ARG and iNOS are induced by inflammatory cues such as cytokines, a key feature that distinguishes them from enzymes that catabolize other AA (9).

In this review, the role played by Trp- and Arg-catabolic pathways in immunological responses in MS is presented.

## Tryptophan and Arginine Metabolism and Its Impact on the Immune Response

L-Tryptophan is an essential AA in mammals derived exclusively from dietary intake or protein catabolism. Evidence suggests that the kynurenine pathway of Trp metabolism is responsible for a broad spectrum of effects, including endogenous regulation of neuronal excitability, initiation of immune tolerance, and synthesis of nicotinamide adenine dinucleotide (NAD^+^), a critical molecule in several biochemical processes (15–17). The broad immunological effects of this pathway are mainly attributable to increased activity of IDO1, which is the key determinant enzyme of Trp metabolism, diverting the process from the production of serotonin towards the production of kynurenines. IDO1-dependent suppression of T cell responses has been proposed as an immunoregulatory pathway implicated in maternal tolerance of allogeneic fetuses during mammalian gestation, as well as in tumor tolerance, and protective regulation in autoimmune disease (9, 15, 18). Studies using IDO1 deficient mice suggest that IDO2 has a limited role (if any) in regulating immunity. Notably, most cell types do not express IDO1 under baseline conditions. In monocytes however, IFN-γ strongly induces IDO1 expression *via* a signal transducer and activator of transcription-1 (STAT-1), and an IFN-regulatory factor (IRF-1)-dependent mechanism (19, 20). Likewise, LPS, TNF-α, Toll-like receptor 9 (TLR9) ligands, IFN-α, TGF-β (via non canonical nuclear factor-κB activity), and CTLA-4 may also induce IDO1 enzymatic activity in cell type- and context-specific fashion, at sites of inflammation (21–25).

Different mechanisms have been proposed to explain immunosuppression observed after IDO1 activation. First, Trp deprivation results in cell-cycle arrest and apoptosis of autoreactive T cells (26, 27). Second, Trp metabolites and Trp depletion act synergistically on CD8^+^ effector T cells, down-regulating expression of the T cell receptor (TCR) CD3 ζ chain, impairing its cytotoxicity activity (28). Third, intermediate metabolites of the kynurenine pathway (L-kynurenine, 3-OH-L-kynurenine, and 3-hydroxyantranilic acid) suppress proliferation and induce preferential apoptosis of Th1 cells (29, 30). Data concerning the contribution of quinolinic acid to T cell regulation is controversial but, this process possibly requires a Trp-depleted microenvironment (30, 31). Fourth, IDO1 is highly expressed in plasmacytoid dendritic cells (DC) which makes them powerful tolerogenic cells (18, 32, 33). Furthermore, this effect on DC blocks the conversion of Treg cells into pro-inflammatory Th17 cells (34). Finally, the main IDO1 catalytic product, kynurenine, has inmunoregulatory effects in the absence of Trp starvation *via* activation of the transcription factor aryl hydrocarbon receptor (AhR).

AhR is a transcription factor activated by several exogenous and endogenous ligands that shapes innate and adaptive immune responses, in a ligand-specific fashion (for review see 35). AhR remains in an inactive state in the cytoplasm, as part of a protein complex. In response to activation by an AhR-ligand this complex is dissociated, and AhR translocates from the cytoplasm to the nucleus, where it controls the transcription of a variety of target genes. In addition, Ahr can also regulate the half-life or activation of other transcription factors, and have genome-wife effects through interactions with transcriptional and epigenetic regulators (for review see (35)). Diverse sources of physiological AhR ligands have been identified so far, including ligands provided by the diet, and enzymatic activities in the host and the commensal flora (36, 37). Kynurenine metabolites can also act as potent AhR ligands, like kynurenic acid, xanthirenic acid acid, and cinnabarinic acid. Other molecules derived from Trp can also provide AhR agonists, like indole acetic acid (38), and 5-hydroxy-Trp (39). The microbial metabolism of dietary Trp also produces additional AhR agonists, such as indole-3 acetic acid, tryptamine, 3-methyl indole (40–42). Different studies have revealed that Trp metabolites exert different actions through AhR and opposite reactions on the immune system, depending on the specific ligand. They can promote differentiation of Th17 at early stages (43), but depending on the stimuli they are exposed to, may favor conversion of Th17 cells to Tr1 cells. AhR also affects Treg cells, boosting differentiation of Tr1 cells in response to IL-27 (44). Likewise, AhR signaling in dendritic cells, decreases pro-inflammatory T cell polarization, favoring anti-inflammatory Treg cell activation (45–47). Trp catabolites such as AhR ligands may therefore provide new insight on how amino acids modulate the immune response.

Effector CD4^+^ T cells and Treg cells require different metabolic programs to develop and exert their function. Aside from amino acid metabolism, IDO1 can alter other metabolic pathways by activating GCN2K, as is the case in CD4^+^ effector T cells, in which IDO1 decreases glucose influx and alters expression and/or phosphorylation of key enzymes involved in glucose metabolism, thereby suppressing aerobic glycolysis (48, 49). IDO1 also starves CD4^+^ T cells of glutamine, another source of energy, through GCN2K activation, by decreasing the expression of both glutaminase isoenzymes (49, 50). These two metabolic events result in reduced production of ATP. Moreover, activation of GCN2K down-regulates enzymes promoting fatty acid synthesis (51). In parallel, activation of AhR, promotes free fatty acid oxidation as an alternative fuel for ATP production, supplying the energy needed for CD4^+^ T-cell survival and proliferation (49). Collectively, these metabolic effects may contribute to IDO1-induced inhibition of CD4^+^ effector T-cell proliferation, and promote differentiation of näive CD4^+^ T cells towards a regulatory phenotype (52–54).

Arginine is a semi essential AA, i.e. required by mammals only under special circumstances, such as for the immune response. Arginine consumption by ARG1, rather than ARG2 or iNOS, plays an important immunoregulatory role in M2 macrophages (55, 56). Murine myeloid cells express ARG1 in response to IL-4, IL-6, IL-13, and TGF-β (57, 58) while in humans, it is constitutively present in the granular compartment of neutrophils, where it becomes activated during inflammation (59). Arginine catabolism induces anergy in tumor-specific effector T cells through a mechanism involving reduced half-life of mRNA encoding the TCR CD3 ζ chain (60, 61). Indeed, suppression of ARG1 activity has immune-mediated anti-tumor effects that inhibit Treg cell proliferation and promote tumor antigen-specific T-cell tolerance (62). In addition, L-Arg starvation restricts T cell activation and proliferation arresting the cell cycle of T-cells in phase G_0_-G_1,_ *via *modulation of cyclin D3 mRNA (63, 64).

Recent studies have demonstrated that ARG1 and IDO1 coexist in DC in the presence of TGF-β. Notably, the induction of ARG1 enzymatic activity by TGF-β is mandatory for subsequent IDO1 upregulation, both in terms of catalytic as well as of signaling mechanisms (65). In addition to the effects of Arg starvation, metabolites originated by its breakdown, particularly polyamines (putrescine, spermidine and spermine) have immunoregulatory properties. Spermidine in particular, can replace TGF-β in the activation of the Src kinase, promoting phosphorylation of IDO1, and thus triggering IDO1 immunoregulatory signaling events in DC. This regulatory circuit responsible for maintenance of long-term immune tolerance, may represent the intersection between the immunometabolic pathways of ARG1 and IDO1 (65).

Cells sense AA levels through at least two different pathways involving serine/threonine kinases: the GCN2K pathway, and the mammalian target of rapamycin (mTOR) pathway. Reduction of AA below the cell threshold causes deacetylation of the corresponding tRNAs. These uncharged tRNAs, present in the neighborhood of the ribosome activate GCN2K (66). Once activated, GCN2K phosphorylates Ser^51^of eukaryotic initiation factor 2α (eIF2α (67)), a factor required for initiation of most eukaryotic translation. These effects result in inhibition of mRNA translation (68). At the same time, and paradoxical fashion, activating transcription factor 4 (ATF4) is translationally up-regulated. ATF4 then binds AA response element, inducing expression of specific target genes that upregulates autophagy and biosynthesis pathways, ultimately allowing cell survival and adaptation to AA insufficiency (68, 69). Although deacylated tRNA is considered the main stimulus for GCN2K activation, recent studies have demonstrated that GCN2K can be alternatively activated by P-stalk complex, a ribosome-associated protein complex, in the absence of tRNA (70). In mammalian cells, mTOR is a critical regulator of cellular growth, proliferation, and differentiation, which exists in two functionally and structurally distinct complexes, mTOR complex 1 (mTORC1) and mTORC2. It has been observed that through Rag GTPases-mediated lysosomal signal transduction, AA levels are critical for mTORC1 activation (71). GCN2K and mTOR are related to each other and jointly assess nutritional deficiency (GCN2K) and adequacy (mTOR). This means that increased AA catabolism (ie activation of IDO1 through inflammatory cytokines) leads to AA depletion, GCN2K activation, and mTOR inactivation (9). However, some studies have shown that mTORC1 is sensitive to a lack of specific amino acids, including leucine, isoleucine, valine, and possibly Arg, but not Trp (72). Arg is therefore capable of stimulating p70S6K activity, an mTORC1 substrate (73), while in other contexts leucine, isoleucine and valine show similar capacity to stimulate 4E-BP1 phosphorylation, a second mTORC1 substrate (74). In line with these observations, inhibition of IDO1 activity in human mixed lymphocyte reactions, prevents GCN2K activation in alloreactive T-cells, but does not affect mTORC1 activity (48). Therefore, IDO1 and mTORC1 pathways sense the absence of amino acids through different mechanisms.

The GCN2K-dependent pathway through the withdrawal of Trp and the presence of Trp catabolites is critical to differentiate naive CD4 + T cells into FOXP3 + Treg cells (28). GCN2K has also been shown to have a key role in activating resting FOXP3 + Treg cells (75). In fact, when these cells are obtained from GCN2K knockout mice, there is no regulatory response of FOXP3 + Treg cells (76). In addition, it was also found that the activation of the AA starvation response dependent on GCN2K can inhibit the differentiation of Th17 in mice and humans (77). In contrast, the mTORC1 pathway regulates metabolic programs to promote the transition of T cells from a resting state to an activated state, and plays a key role in Th17 cell differentiation (78, 79). At the same time, mTORC2 regulates the ability of Th cells to differentiate into Th2 cells (80, 81). Although the contribution of mTORC2 *in vivo* is not as significant as that of mTORC1, both mTORC1 and mTORC2 seem to interfere with the differentiation and function of Treg cells (82). The excessive activation of the mTORC1 pathway is related to the decreased expression of the 44- and 47-KDs forms of FOXP3 and the increased expression of the cell cycle inhibitor p27kip1. Although mTORC1 drives a pro-inflammatory response in adaptive immunity, it may have an anti-inflammatory effect on the innate immunity, promoting polarization of macrophages M1 to macrophages M2 (83).

## Amino Acid Catabolism in Multiple Sclerosis Affects Immune Homeostasis

Several lines of evidence indicate that IDO1 is a negative endogenous regulator of CNS inflammation. In experimental autoimmune encephalomyelitis (EAE), an animal model for MS, IDO1 activity is increased in the spleen during pre-clinical phases, and IDO1-positive microglia and macrophages have been detected in brain and spinal cord at symptom onset, correlating with disease severity. Furthermore, inhibition of IDO1 using 1-methyl-Trp exacerbates disease scores, revealing IDO1 expression influence over immune regulation. These findings suggest that IDO1 is induced by IFN-γ from encephalitogenic Th1 cells, thereby participating in a negative feedback loop contributing to inflammation auto-limitation (84). Likewise, IDO1-deficient mice showed clinical EAE exacerbation with an increase in Th1 and Th17 cells, as well as reduction of Treg cells (11). In contrast, administration of the downstream Trp metabolite 3-HAA, inhibited effector Th1 and Th17 cells and decreased EAE clinical scores by promoting Treg cells and by up-regulating TGF-β production by DC (11). The presence of IDO1 positive microglia and macrophages in EAE suggests that IDO1 activation might be a concomitant self-protective response in EAE, designed to counteract the effects of autoreactive lymphocytes *via* Trp depletion and elevation of downstream kynurenines (84). Similarly, administration of cinnabarinic acid, a metabolite of the kynurenine pathway, which acts as an agonist of the type-4 metabotropic glutamate receptor (mGlu4), significantly protects EAE animals, by boosting an immune response dominated by the presence of Treg cells at the expense of Th17 cells. In addition, administration of cinnabarinic acid increased its endogenous production by lymphocytes, suggesting the existence of a positive feedback loop, sustaining immune tolerance (85). Supporting these findings, knockout mice lacking mGlu4 were markedly vulnerable to EAE (85, 86).

Although the evidence sustaining the significance of AA catabolism in immune regulation is growing, the importance of this pathway in MS patients is still very small and most of them are inferred from animal models. Our group recently demonstrated that compared with healthy controls and patients with other inflammatory neurological diseases, the expression and activity of IDO1 and ARG1 in MS patients are significantly reduced, mainly in monocytes, and, to a minor degree in Trig cells and Th1 (87). Interestingly, faulty IDO1 activity in MS patients is related to the differential distribution of IDO1 rs7820268 single nucleotide polymorphism (88). When assessing the functional consequences of reduced AA catabolism, we observed that high levels of Tarp and Argo were associated with a significant down-regulation of GCN2K, and a strong up-regulation of mTORC1 expression and activity, but not of mTORC2. In turn, this leads to a decrease in the number of Trig cells, an increase in the proliferation of MBP self-reactive T cells, and an increase in the production of pro-inflammatory cytokines (87).

Studies evaluating kynurenin levels in CSF and plasma from MS patients have shown conflicting results (89–92). Discrepancies observed may result from evaluation of patients at different stages of the disease. A study analyzing changes in IDO1 expression and activity in peripheral blood mononuclear cells (PBMC) of relapsing remitting MS patients, showed high IDO1 expression during relapsing phases but not during remission, suggesting activation of Trp catabolism may be associated with disease activity, perhaps representing a self-protective response to control the autoimmune response, similar to the one described in EAE (93).

In addition to the kynurenine pathway, Trp is metabolized through an alternative metabolic route that produces sequentially serotonin, N-acetylserotonin (NAS), and melatonin. N-acetylserotonin has been shown to possess antioxidants, anti-inflammatory, and neuroprotective properties in EAE, activating the TrKB receptor (94, 95). Interestingly, NAS directly binds IDO1 and acts as a positive allosteric modulator of the enzyme both *in vitro* and *in vivo*. As a result, increased kynurenine will activate the AhR and consequently anti-inflammatory and immunoregulatory effects (88).

In addition, studies from our group have demonstrated that melatonin is an additional regulator of the immune response in MS. Treatment with melatonin ameliorates disease in EAE, and interferes with the differentiation of human and mouse T cells. Melatonin induces the expression of the repressor transcription factor Nfil3, blocking the differentiation of Th17 cells, and promotes Tr1 differentiation of protective Tr1 cells by activating Erk1/2 signaling and the transcription of the IL-10 promoter ROR-α (96).

Although IDO1 activation plays an important role in the development and maintenance of immunological tolerance, metabolites produced along the kynurenine pathway can also have neurotoxic effects within the CNS. Quinolinic acid, and 3-hydrokynurenine are the most important examples, causing acute neuronal death, and chronic CNS dysfunction (16, 89, 97). Concentrations of these metabolites, synthesized mainly in macrophages, are increased to neurotoxic levels in the spinal cord and lower brainstem of rats with EAE (97). Quinolinic acid toxicity could be attributed to several different mechanisms: i) is a weak but specific agonist of the N-methyl-D-aspartate receptor; ii) inhibits glutamate uptake by astrocytes; iii) generates reactive oxygen toxic intermediates; iv) mediates depletion of endogenous anti-oxidants and peroxidation of lipid molecules; and v) induces chemokine ligands (CCL5, and MIP-1) in astrocytes, and chemokine receptors (CXCR4,CXCR6, CCR3, and CCR5), which promote leukocyte attraction across the blood brain barrier (16, 89, 97–99). In contrast to macrophages, synthesis of quinolinic acid is very heterogeneous in different microglia cell populations, and significantly lower compared to macrophages (100). These differences may be due to different kynurenin pathway enzymes expression in both cell types (101). Under pathological conditions with blood-brain-barrier breakdown and important leukocytes infiltration, quinolinic acid levels in the CNS may substantially derived from peripheral macrophages (102). Meanwhile, toxic effects of 3-hydrokynurenine are mediated through the production of free radicals (103). Some of these considerations are especially important in MS, since some kynurenine pathway products, particularly quinolinic acid, are toxic not only for neurons, but also for oligodendrocytes, thus contributing to the process of demyelination (104, 105). By contrast, picolinic acid and kynurenic acid synthesized in neurons and astrocytes, exert neuroprotective effects by acting as endogenous glutamate receptor antagonists and free radical scavengers (106). Interestingly, recent data provides evidence that, over time, the initially suppressive T-cell effect mediated by IDO1, changes to a more chronic form of kynurenine pathway activation that leads to MS progression, through production of quinolinic acid by infiltrating macrophages. In addition, as 3-hydrokynurenine is known to potentiate quinolinic acid-induced toxicity, the higher levels of 3-hydrokynurenine in secondary progressive MS and primary progressive MS patients observed in this study, might be relevant to the neurodegenerative process observed in MS (92). Thus, dysregulation of one or more enzymatic steps in the kynurenine pathway, may shift the balance towards production of either neurotoxic or neuroprotective metabolites (107).

Of note, in addition to decreased catabolism of Trp and Arg, free intracellular AA assays in PBMC from MS patients, showed significant increase in Leu, Ile, and Glm compared to healthy subjects (87). Interestingly, Glm in combination with Leu activates mammalian mTORC1 by enhancing Glm metabolism, promoting cell growth and inhibiting autophagy (108). Moreover, blocking Leu and glucose metabolism inhibits mTORC1 activation, which in turn promotes the induction of anergy in Th1 cells (109). Likewise, loss of the Leu transporter limits T cell activation and effector maturation owing to impairment in mTORC1 activity (110). Collectively, these observations indicate that control of intracellular AA availability, could regulate immune responses through different mechanisms.

## Conclusion and Future Perspectives

**Figure 1:**
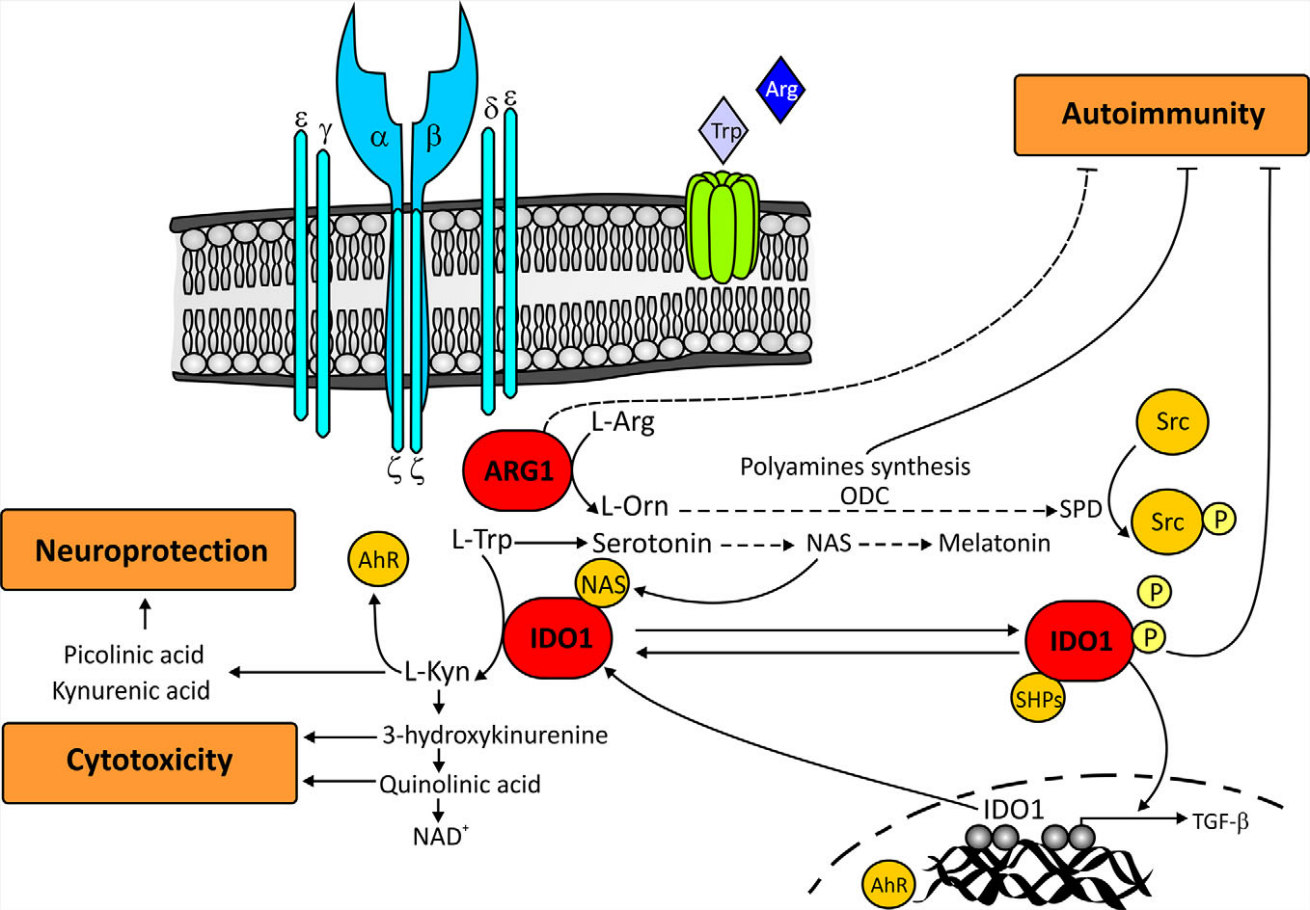
Main IDO1 and ARG1-mediated pathways that regulate autoimmunity, cytotoxicity, and neuroprotection. The increase in ARG1 activity transforms L-Arginine into L-Ornithine, which is subsequently metabolized in the polyamine synthesis pathway. This metabolic pathway gives rise to spermine which, through the phosphorylation of Src kinase, facilitates the phosphorylation of IDO1. Once phosphorylated, IDO1 recruits tyrosine phosphatases, and promotes the upregulation of TGF-β and IDO1 coding genes. For its part, IDO1 catalyzes the conversion of L-Tryptophan into L-kynurenine which activates the aryl hydrocarbon receptor. Aryl hydrocarbon receptor further induces IDO1 expression and contributes to the production of polyamines through the stimulation of L-Ornithine metabolism. Together, the polyamine and IDO1 pathways, and probably ARG1, are capable of inhibiting the autoimmune response. L-Tryptophan through the kynurenine pathway is also capable of generating downstream metabolites both cytotoxic (3-hydroxykynurenine and quinolinic acid), and neuroprotective (picolinic acid and kynurenic acid). In addition to the Kynurenine pathway, Tryptophan is sequentially metabolized to serotonin, N-acetylserotonin, and melatonin. N-acetylserotonin acts as an allosteric modulator of IDO1, while melatonin contributes to the regulation of the autoimmune response by blocking the differentiation of Th17 cells and boosting the generation of protective Tr1 cells. For reasons of clarity in the figure, some of the participating routes have been omitted. Ahr, aryl hidrocarbon receptor; ARG1, Arginase1; IDO1, indoleamine 2,3-dioxygenase; L-Arg, L-Arginine; L-Kyn, L-Kynurenine; L-Orn, L-Ornithine; L-Trp, L-Tryptophan; NAD, Nicotinamide adenine dinucleotide; NAS, N-acetylserotonin; ODC, ornithine decarboxylase; SHPs, tyrosine phosphatases; SPD, spermine; Src, Src kinase; TGF-β, Transforming growth factor-β.

Since AA consumption affects immunity in varied and often diametrically opposite ways, elucidation of the diverse mechanisms through which pathways catabolizing AA influence immune responses is a challenging task. The main pathways mediated by IDO1 and ARG1, which modulate the autoimmune response, as well as mechanisms of cytotoxicity and neuroprotection are summarized in the **Figure 1**. Both Trp and Arg metabolism represent critical immune checkpoint mechanisms affecting autoimmune disorders, neoplasms, and transplants. With the discovery of so many new pathway components, understanding the interplay between positive and negative regulators is of great interest. Many issues and controversies remain related to their precise function in immune regulation, neuroprotection, and excitotoxicity. Nevertheless, the development of emerging opportunities to target biochemical pathways regulating AA as potential strategies to treat different diseases, has become an extremely important topic for research. Future treatment strategies could include: catabolism inhibition of certain AA to enhance immunity, increasing AA catabolism to suppress hyper-immunity, selectively inducing IDO1, use of inhibitors of the GCN2K pathway, or use of the immuno-suppressive Trp metabolites, among others.

## Author Contributions

The author confirms being the sole contributor of this work and has approved it for publication.

## Funding

This work was supported by an internal grant from Fleni (JC).

## Conflict of Interest

The author declares that the research was conducted in the absence of any commercial or financial relationships that could be construed as a potential conflict of interest.
